# Effect of Different Thawing Rates on Post-Thaw Viability, Kinematic Parameters and Chromatin Structure of Buffalo (Bubalus bubalis) Spermatozoa

**Published:** 2013-02-20

**Authors:** Abdolreza Rastegarnia, Abdolhossein Shahverdi, Tohid Rezaei Topraggaleh, Bita Ebrahimi, Vahid Shafipour

**Affiliations:** 1. Department of Clinical Science, Faculty of Veterinary Medicine, Urmia Branch, Islamic Azad University, Urmia, Iran; 2. Department of Embryology at Reproductive Biomedicine Research Center, Royan Institute for Reproductive Biomedicine, ACECR, Tehran, Iran; 3. Buffalo Breeding and Training Extension Center, Jabal, Urmia, Iran

**Keywords:** Thawing Rate, Motility, Chromatin structure, Buffalo

## Abstract

**Objective:**

The aim of the present study was to evaluate three thawing rates on the post thaw motility, viability and chromatin structure of buffalo semen frozen in 0.5-ml straws.

**Materials and Methods:**

In this experimental study semen was collected with artificial vagina (42℃) from four buffalo bulls.Split pooled ejaculates (n=4) were extended at 37℃ with a Bioxcell® extender. Semen was cooled to 4℃ within 2 hours, equilibrated at 4℃ for 4 hours, then filled in 0.5 ml French straws, and frozen in programmable cell freezer before plunging into liquid nitrogen. Straws were thawed at water bath temperatures of 37, 50 or 70℃ for 30, 15 and 6 seconds, respectively. Semen was incubated at 37℃ for 2 hours and evaluated for post thaw motility, viability, acrosomal and DNA integrity of spermatozoa. Analysis of variance (ANOVA) was used for comparisons of means. When the ANOVA test showed statistical differences, the mean of the treatments were compared using Duncan’s multiple range tests.

**Results:**

The initial postthaw motility (0 hour) averaged 62.7 ± 7.2%, 73.1 ± 9.77%, and 74.9 ± 8.58% for the three thaw rates, respectively. Kinematic parameters such as average path velocity, linearity and beat/cross frequency in the thaw rate of 70℃ for 6 seconds were superior to other rates studied (p<0.05). After 2 hours of incubation, proportions of progressive motility and Kinematic parameters decreased in all groups (p>0.05). A positive correlation was detected between sperm motility and thawing rate after two hours incubation times. The percentage of viable spermatozoa and spermatozoa with an intact acrosome and plasma membrane integrity were not different between the groups of samples thawed at different temperatures (p>0.05). The percentage of spermatozoa with chromatin dispersion forthe thaw rate of 70℃ for 6 seconds was significantly higher than for the to other rates studied (p< 0.05). In contrast with motility and viability, the DNA integrity of post thaw spermatozoa remained unaffected during 2 hours incubation.

**Conclusion:**

The post thaw motility and kinematic parameters of buffalo spermatozoa were significantly improved immediately after thawing by increasing the thawing rate from 37℃ in 30 seconds to70℃ in 6 seconds. However, this relative advantage had disappeared after incubation in a water bath at 37℃ for two hours.A thaw rate of 70℃ for 6 seconds was associated with higher chromatin dispersion than the other thaw rates studied. Sperm thawing over at 50 degrees could be safely used to improve motility recovery after sperm cryopreservation in buffalo bulls.

## Introduction

High viability and motility of spermatozoa are important factors for successful artificial insemination (AI) because a significant correlation between post-thawing sperm viability and subsequentconception rate has been reported (1, 2). There are numerous factors that may affect the motility, plasma membrane integrity and viability of buffalo bull semen during storage e.g. type of extender, permeable and non-permeable cryoprotectants, packaging system or freezing and thawing time (3).

The rate of thawing significantly affects sperm survival, and the appropriate thawing rate is thought to be influenced by numerous factors in the cryopreservation procedure such as type of extender, glycerol concentration, packaging method and freezing rate (4). Many different methods for thawing semen in straws have been recommended including a variety of water bath temperatures and thawing periods, shirt pocket thawing, air-thawing in the palms of the hands, and thawing in the cow (5). Overall, unless specific recommendations are given it is recommended that, buffalo semen frozen in straws, irrespective of extender type and cooling rate, should be thawed in a water bath at 33-35℃ for 30-40 seconds (6, 7). However, a number of studies have shown that thawing temperatures as high as 60-80℃ could further improve post-thaw motility (7-12). Although the use of such a high temperature is far from being a practical method of thawing the straws, especially in field conditions, it appears to cause a lower degree of cellular damage; however, the magnitude of this effect is still unknown.In the freeze-thaw procedure, the warming phase is just as important to the survival of spermatozoa as thefreezing phase. Spermatozoa that have survived cooling to -196℃ still face the challenge of warming andthawing, and thus must traverse twice the criticaltemperature zone i.e., from -5 to -50℃ (13). A practical thaw for bull spermatozoa, recommended by most AI organizations, is as 35℃ water bath for at least 30 seconds (10, 14, 15). It has been shown that an increase in post-thaw viability will result in increased fertility of the semen (5).

Most of the previous studies designed to evaluate thawing procedures for Buffalo spermatozoa were based on the subjective microscopic assessment of post-thaw sperm motility, morphology or membrane integrity (8, 10).

The present study used flowcytometry and Computer- Assisted Semen Analysis (CASA) systems which provide more objective and precise measurements of the functional and structural characteristics of spermatozoa. The application of these new technologies to semen analysis could allow us to re-evaluate some the controversial aspects of existing cryopreservation protocols, such as whether there is real advantage in using thawing temperatures higher than 37℃. Therefore, the main purposes of the present study were to evaluate two faster thawing rates than the one usually recommended for buffalo spermatozoa frozen in straws. The two thawing rates chosen were: 50℃ for 15 seconds, or 70℃ for 6 seconds in a water bath. The recommended thawing procedure (placing the straws into a water bath at 37℃ for 30 seconds) was used as the control.

## Materials and Methods

### Semen collection and freezing

The experiment was conducted at the Buffalo Breeding and Extension Training Center, Urmia, West Azerbaijan, Iran Oct-Dec 2011. Four adult buffalo bulls (*Bubalus bubalis*) of known fertility and similar age (4-5 years) were used for semen collection. Two ejaculates from each bull were collected in the artificial vagina (at 42℃) at 10 minutes intervals weekly for4 weeks (replicates). Ejaculated semen from each bull (4 ejaculates/bull) was immediately transferred to the laboratory. The progressive motility of the Sperm was determined microscopically (-400; Olympus BX20, Tokyo, Japan) and sperm concentration was determined by digital photometer (IMV, France). To eliminate individual differences, semen samples from the four bulls were pooled. Bioxcell® was prepared according to the manufacturer’s instructions (IMV, France). Split pooled ejaculates, possessing more than 70% visual sperm motility were diluted with Bioxcell® extender at a concentration of 50 × 10^6^ motile spermatozoa ml ^-1^ at 37℃. Diluted semen was cooled to 4℃ in 2 hours, equilibrated for 4 hours at 4℃, decanted into 0.5 ml French straws (IMV, France) with a suction pump at 4℃ in a cold cabinet unit (IMV, France) and placed in liquid nitrogen vapors, 4 cm above the level of liquid nitrogen for 10 minutes. Straws were then plunged into the liquid nitrogen (-196℃) and stored until examination.

### Thawing procedures

Four straws from each bull were respectively thawed at the following three rates: i.70℃ for 6 seconds; ii. 50℃ for 15 seconds, and iii. control:

37℃ for 30 seconds. Thawing was done by placing the straws in a water bath at the proper temperature. Immediately after thawing, the content of each straw was emptied in a 5-ml Falcon tube at 37℃. The sperm suspension was kept at 37℃ during post-thaw incubation.

### Semen evaluation

Semen analysis was conducted in the Department of Embryology and Reproductive Medicine Research Center of the Royan Institute.

### Motility

An aliquot of post thaw semen (5µL) was placed on a prewarmed (37℃) Makler chamber (depth 10 µm) and analyzed for sperm motion characteristics using a computer-assisted sperm analyzer (Sperm Class Analyzer, Microptic; Barcelona, Spain). The CASA-derived motility characteristics were analyzed immediately after thawing (0 hour) and after two hours of incubation at 37℃. Three microscopic fields were analyzed in each sample using a phasecontrast microscope (Nikon, Tokyo, Japan) supplied with a prewarmed stage at 37℃ and at × 100 magnification. The total number of spermatozoa analyzed per sample ranged between 100 and 200. Objects incorrectly identified as spermatozoa were minimized on the monitor by using the playback function. Total motility was defined as the percentage of spermatozoa with mean velocity (VAP, µm/s) above 10 µm/s. The CASA derived motility characteristics studied were percentage of motility and progressive motility, straight-line velocity (STR), curvilinear velocity (VCL, µm/s), lateral head displacement (LHD, µm), linearity (LIN, %; VSL/VCL × 100), and straightness (STR, %; VSL/VAP × 100), Lateral Admplitude ( ALH, µm) and Beat Frequency (HZ), (16).

### Sperm viability

Eosin-nigrosin (Eo Nig) staining was used to evaluate sperm viability (17). After thawing, one drop of the semen was placed on a tempered glass slide, which was mixed with one drop of Eo Nig solution (0.2 g of eosin and 2g of nigrosin were dissolved in a buffered saline solution [153 mM NaCl and 9.65 mM NaH_2_PO_4_, pH=7.4], mixed for 2 hours at room temperature and filtered to obtain the staining media). The mixture was smeared on the glass slide and allowed to air dry. One hundred spermatozoa were evaluated in at least five different fields in each smear under a light microscope. Eosin penetrates in non-viable cells, which appear red. Nigrosin offers a dark background facilitating the detection of viable, non-stained cells.

### Sperm plasma membrane integrity

Sperm plasma membrane integrity (PMI) was determined using the hypo-osmotic swelling (HOS) assay. HOS solution consisted of 0.73g sodium citrate and 1.35 g fructose dissolved in100 ml distilled water (osmotic pressure-190 osmoleKg-1). To assess the sperm tail plasma membrane integrity, semen (50 µl) was mixed with 500 µl of HOS solution and incubated for 30 minutes at 37℃ before examination under a phase contrast microscope (X400; Olympus BX20, Tokyo, Japan). Two hundred spermatozoa were assessed for swelling. The swollen spermatozoa,characterized by coiling of the tail, were considered to have an intact plasma membrane (18).

### Normal Acrosomes

To assess sperm acrosomal integrity, 100 µl of semen sample was fixed in 500 µl of 1% formal citrate (2.9 g tri-sodium citrate dihydrate, 1 ml of 37% solution of formaldehyde, dissolved in 100 ml of distilled water) for a 15 minutes. One hundred spermatozoa were examined with a phase contrast microscope (X1000; Olympus BX20, Tokyo, Japan) under oil immersion. A normal acrosome was characterized by a normal apical ridge (NAR), (19).

### Assessment of DNA integrity

Chromatin stability was assessed by using the SCSA (Sperm Chromatin Structure Assay) technique. This technique is based on the susceptibility of the sperm DNA to acid induced denaturation in situ shown by the meta chromatic shift in Acridine Orange (AO) stain from green (dsDNA) to red (ssDNA) fluorescence depending on the degree of DNA denaturation (20). After thawing at the three thawing rates previously specified, samples were diluted with Tris-Null-EDTA (TNE) buffer (0.01 m Tris-HCl, 0.15 m NaCl, 1 mm EDTA, pH=7.4) in cryotubes, at a final sperm concentration of 20 × 10^6^ cells/ml. A 100µl aliquot of this suspension was mixed with 200µl of a detergent/acid solution (0.1% v/v Triton X-100 in 0.08MHCl, 0.15 MNaCl). After 30 seconds, 0.6 ml of AO solution (6 µg/ml of AO in 0.15 M NaCl, 1mM EDTA, 0.2 M Na_2_HPO_4_, 0.1M citric acid, pH=6.0) was added to the sample and the cells were subjected to flow cytometry after 30 minutes incubation at room temperature (21).

### Flow cytometer analysis

Flow cytometric analysis was performed using FACS Calibur (BD, Immunocyttometry Systems, San Jose, CA, USA) with an air-cooled argon laser operated at 488 nm excitation and 15mW. For the AO assay, the green fluorescence (intact DNA) detected by the FL-1 detector (515/45-nm band-pass filter) was compared with red fluorescence (single-stranded DNA) detected by the FL-3 detector (640 nm longpass filter) after gating out non-sperm and aggregated events. Ten thousand sperm cells were acquired and analyzed in each sample at the rate of 1000 events per second and analyzed further with cytoflogic software (Cyflogic version 1.2.1).

### Statistical analysis

Each treatment was replicated six times. For each replicate, four straws were thawed and pooled for evaluation of sperm parameters. For assessment of DNA fragmentation each treatment consisted of at least three replicates. ANOVA was used for comparisons of means. When the ANOVA test showed statistical differences, the mean of the treatments were compared using Duncan’s multiple range tests. All the statistical analyses were performed using the SAS software (Version 9.0, SAS Institute Inc., USA), and differences were considered significant at p< 0.05 level. Results are presented as mean ± standard deviation.

## Results

### Motility

The thawing rate had significant effects on sperm motility as determined subjectively. At the onset of incubation, proportions of motile spermatozoa and progressively motile spermatozoa thawed at 37℃ for 30 seconds, at 50℃ for 15 seconds or at 70℃ for 6 seconds, were 62.7 ± 7.2%, 25.4 ± 4.9% , 73.11 ± 9.7% 38.7 ± 6.73% and 74.85 ± 8.6%, 41.34 ± 9.43%, respectively (p<0.05, Table 1). Kinematic parameters such as VAP, LIN and BCF in the thaw regime 70℃ for 6 seconds were superior compared to the other regimes studied. After two hours of incubation at 37℃, proportions of motile and progressively motile spermatozoa decreased to 57.9 ± 5.57%, 8.9 ± 2.6%, 61.59 ± 7.49%, 7.65 ± 5.46% and 62.61 ± 9.7%, 6.83 ± 3.2% for samples thawed at 37℃ for 30 seconds, 50℃ for 15 seconds and at 70℃ for 6 seconds, respectively (p>0.05, Table 2). Kinematic parameters also decreased after two hours incubation at 37℃, but were notdifferent between the groups.

**Table 1:** Mean (± SD) motility characteristics for the buffalo bull semen samples thawed at different time, after 0 hour of incubation at 37℃


Thawing rate (s)			
	37	50	70

Motility (%)	62.7 ± 7.23^a^	73.1 ± 9.77^b^	74.8 ± 8.58^b^
PM (%)	25.4 ± 4.94^b^	38.7 ± 6.73^a^	41.3 ± 9.43^a^
VCL (m/s)	40.1 ± 5.25^b^	70.5 ± 9.31^a^	73.8 ± 16.5^a^
VSL (m/s)	15.9 ± 6.28^b^	35.1 ± 7.66^a^	35.1 ± 7.56^a^
VAP (m/s)	21.6 ± 7.09^b^	42.3 ± 8.80^a^	42.4 ± 8.84^a^
LIN (%)	35.6 ± 5.98^b^	48.7 ± 4.77^a^	47.8 ± 6.45^a^
STR (%)	69.8 ± 7.78^b^	82.9 ± 1.94^a^	79.8 ± 9.18^a^
ALH (m)	1.81 ± 0.36^b^	2.61 ± 0.38^a^	2.68 ± 0.55^a^
BCF (Hz)	14.3 ± 4.35^b^	13.9 ± 0.46^b^	18.1 ± 3.02^a^


PM: progressive motile spermatozoa, VSL; straight path velocity, VCL; curvilinear velocity, VAP; average path velocity, LIN; linearity; STR; straightness, ALH; amplitude of the lateral movement of the head and BCF; beat frequency. a, b; Values in the same row with different superscripts differ significantly (p<0.05).

**Table 2:** Mean (± SD) motility characteristics for the buffalo bull semen samples thawed at different time, after 2 hours of incubation at 37℃


Thawing rate (s)			
	37	50	70

Motility (%)	57.9 ± 5.57	61.5 ± 7.49	62.6 ± 9.77
PM (%)	8.98 ± 2.60	7.65 ± 5.46	6.83 ± 3.20
VCL (m/s)	25.6 ± 4.62	22.9 ± 4.52	23.6 ± 3.09
VSL (m/s)	5.62 ± 2.35	5.19 ± 3.97	4.47 ± 2.72
VAP (m/s)	9.17 ± 2.86	8.45 ± 4.44	7.64 ± 3.15
LIN (%)	21.3 ± 6.00	20.7 ± 11.1	18.8 ± 11.3
STR (%)	59.8 ± 6.36	55.9 ± 12.7	52.5 ± 13.9
ALH (m)	1.65 ± 0.36	1.27 ± 0.14	1.41 ± 0.23
BCF (Hz)	5.88 ± 3.90	5.46 ± 4.97	4.75 ± 4.09


PM; progressive motile spermatozoa; VSL; straight path velocity, VCL; curvilinear velocity; VAP; average path velocity, LIN; linearity, STR; straightness, ALH; amplitude of the lateral movement of the head and BCF; beat frequency.

### Comparison of post-thaw sperm viability, plasma membrane integrity and acrosomal ridge for different thawing rate

The data on viability, plasma membrane integrity and percentage of buffalo bull spermatozoa with a normal apical ridgeare given in table 3. Immediately after thawing, the proportion of viable post-thaw sperm was (76.3 ± 1.5, 79.7 ± 2.5, 81.6 ± 3.8), plasma membrane integrity (61.3 ± 1.5, 58.2 ± 2.1, 63.6 ± 3.8) and normal apical ridge (79.9 ± 1.2, 82.3 ± 0.9, 80.3 ± 1.2) remained similar (p>0.05) for all three thaw regimes.After incubation at 37℃ for two hours spermviability (66.3 ± 1.51%, 62.7 ± 2.55% and 65.5 ± 3.82%),plasma membrane integrity (54.1 ± 6.33%, 49.6 ± 3.14% and 52.1 ± 4.33%) and the percentage of spermatozoa with a normal acrosomal ridge(57.5 ± 1.5%, 52.1 ± 3.1%, 53.6 ± 4.8%) remained similar between the groups, although the levels dramatically decreased for samples thawed at 37℃ for 30 seconds, 50℃ for 15 seconds and at 70℃ for 6 seconds, respectively. The thawing rate as well as the interaction between thawing rate and incubation time had not significant effects.

### Comparison of post-thaw sperm DNA damage in different thawing rate

**Fig 1:**
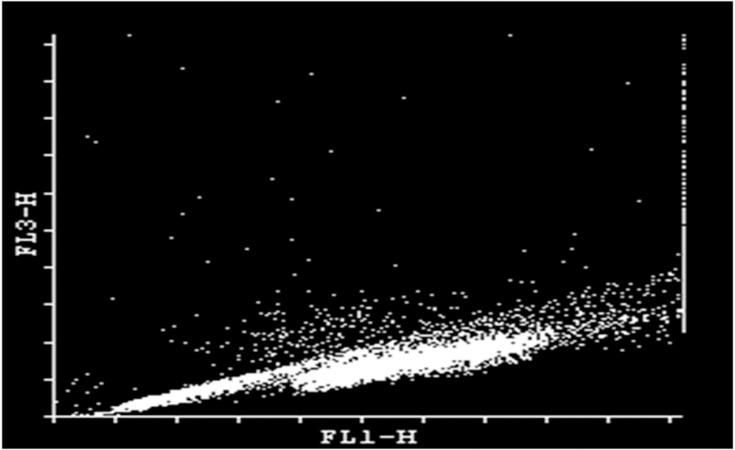
Example of SCSA cytogram of individual buffalo post thaw sperm cells. Each cell’s position is based on theamount of native DNA satiability(green fluorescence; FL1) vs. fragmented DNA (red fluorescence; FL3).

Chromatin damage in each sperm was quantified by red fluorescence. Each semen sample contained percentage of mature cells with non-detectable (main population of spermatozoa in semen) and detectable (percentage of mature spermatozoa with increased chromatin damage) DNA damage.Each cell’s position is based on the amount of native DNA satiability (green fluorescence; FL1) vs. fragmented DNA (red fluorescence; FL3), (Fig 1). After the thawing process, the percentage of spermatozoa with damaged DNA in the sperm thawed at 70℃ for 6 seconds was significantly higher the other thaw rates studied (p< 0.05). The overall mean DNA damage was (5.02 ± 1.3, 6.88 ± 0.89, 7.57 ± 0.62) for samples thawed at 35℃, 50℃ and 70℃, respectively). In contrast with motility and viability the DNA integrity of post thaw spermatozoa remained unaffected during two hours incubation, it was 5.236 ± 1.4 %, 7.27 ± 1.48% and 7.91 ± 0.98%, respectively, for samples thawed at 37℃, 50℃ or 70℃) (p> 0.05, Table 3).

**Table 3:** Mean (± SD) viability, plasma membrane integrity (PMI), normal apical ridge (NAR) and DNA damage for the buffalo bull semen samples thawed at different time after 0 hour and 2 hours of incubation at 37 ℃


Thawing rate (s)						
	37		50		70	

Incubation time (hours)	0	2	0	2	0	2
Viability (%)	76.3 ± 1.58^a^	66.3 ± 1.51^b^	79.7 ± 2 54^a^	62.7 ± 2. 55^b^	81.6 ± 3. 81^a^	65.6 ± 3.82^b^
PMI (%)	61.3 ± 1.51^a^	54.1 ± 6.33^b^	58.0 ± 2.11^a^	49.6 ± 3.14^b^	63.6 ± 3.85^a^	52.1 ± 4.33^b^
NAR (%)	79.7 ± 1.24^a^	57.5 ± 5.55^b^	82.3 ± 0.91^a^	52.1 ± 3.15^a^	80.3 ± 1.24^a^	53.6 ± 4.80^b^
DNA damage (%)	5.02 ± 1.35^a^	5.23 ± 1.47^a^	6.88 ± 0.89^a^	7.27 ± 1.48^a^	7.57 ± 0.62^b^	7.91 ± 0.98^b^


a,b; Values in the same row with different superscripts differ significantly (p<0.05).

## Discussion

In the present study, the proportions of spermatozoa characterized by progressive motility and kinematic parameters such as linear motility were significantly increased by thawing at 50℃ for 15 seconds and 70℃ for 6 seconds, compared with the normal, control rate of 37℃ for 30 seconds.

The present study also recorded a linear trend of increased sperm velocities (VSL and VAP) and LIN after thawing at 70℃ for 6 seconds. The pattern of sperm motion reflects the biochemical environment and physical conditions imposed on spermatozoa. Freezing and thawing cause considerable damage to motion characteristics (visual or computerized motility, and curvilinear velocity), plasma membrane integrity, and the acrosomal ridge of buffalo spermatozoa (22). Post-thaw decline in curvilinear velocity could be due to cryoin juries to the mitochondrial apparatus and axoneme of spermatozoa (2, 16).

Rasoul et al, (22) reported although the early stages of cryopreservation (dilution, cooling, and equilibration) did not affect overall motility, freezing and thawing changed the sophisticated or fine parameters of sperm motion in buffalo bull.Such changes increase the levels of intracellular calcium resulting in increased circular motility and lateral head displacement of spermatozoa (16, 22, 23). However, another study in bull sperm observed no differences in overall post thaw motility or plasma membrane integrity and normal acrosomal ridge between the three thawing methods and higher proportions of spermatozoa with fast and progressive movement were observed after two hours of postthaw incubation when the thawing was at the faster rates (35℃/40 seconds: 8.3%, 50 ℃/15 seconds: 18.1% and 70 ℃/5 seconds: 16.5%) (24).

These results are in concordance with reports by C´ordova-Izquierdo (2006), who demonstrated that in boars thawing straws at 42℃, 40 seconds significantly reduced motility compared to straws thawed at 50℃, 40 seconds and normal acrosomal ridge, penetration, monospermy and polyspermy were not different between the two groups of samples thawed at different temperatures (25).

A practical thaw for buffalo bull spermatozoa, recommended by most AI organizations, is in a 35℃ water bath for at least 30 seconds (3, 6). A variety of studies hadevaluated a range of different thawing rates for buffalo bull semen frozen in straws. The positive correlation between sperm motility and thawing rate recorded in the present study is in line with Ahmad et al. (8) who generally concluded that the more rapid thawing rates result in better sperm motility and acrosomal integrity (8). For cryopreservation of buffalo spermatozoa in Tris-based extender, analyzed Narasimha Rao et al. (7) tested two thawing rates (37℃ for 30 seconds and 75℃ for 9 seconds) (7). They concluded that the best value for post-thaw motility was observed for semen thawed at 37˚ for 30 seconds. The effect of thawing rates (40℃ for 60 seconds, 60℃ for 15 seconds and 80℃ for 5 seconds) on post-thaw motility of buffalo spermatozoa cryopreserved in Trisbased extender. has shown that thawing at 60℃ for 15 seconds yielded a higher sperm motility compared to other rates (9). In another study, Dhami et al. (10) determined the thawing rates for buffalo semen. The thawing rates investigated were 4℃ for 5 minutes, 40℃ for 1 minutes or 60℃ for 15 seconds. They concluded that thawing of semen at 60℃ for 15 seconds yielded high post-thawing spermatozoa recovery and longevity (10). Sukhato et al. (26) determined the effect of thawing rates on motility and acrosome integrity of buffalo spermatozoa. Thawing of spermatozoa was performed at the rate of (rapid) 1000℃ or (slow) 200℃ ⁄ minute. They concluded that rapid thawing was superior to slow warming (26).

The thawing effect depends on whether the rate ofcooling has been sufficiently high to induce intracellular freezing, or low enough to produce cell dehydration. In the former case, fast thawing is required to prevent recrystallization of any intracellular ice present in the spermatozoa. Spermatozoa thawed at a fast rate may also be exposed for a shorter time to the concentrated solute and cryoprotectant-glycerol, and the restoration of the intracellular and extracellular equilibrium is more rapid than for slow thawing (18, 27). Also leaving straws in high temperatures for too long time may result in pH fluctuation and subsequently protein denaturation and cell death (16).

In this study, the sperm plasma membrane integrity was assessed through the HOS test that has been recognized as a reliable procedure for the evaluation of the functional status of the sperm plasma membrane. During cryopreservation, sperm plasma membranes are destabilized due to low temperature and high salt concentration (28). The HOS is a stress assay to assess the functional integrity of the sperm plasma membrane under low osmotic conditions (18).

According to results in the present study, there were no differences in plasma membrane integrity between the thawing regimes. (p>0.05, Table 3). After 2 hours of incubation, total sperm plasma membrane integrity decreased for the three treatments (p<0.05). Similar results were found by Rasoul et al. (22) that the plasma membrane integrity of spermatozoa was reduced due to incubation after freezing and thawing.

The presence of an acrosomal cap is important for the fertilization process and has been highly related with fertilityof frozen buffalo semen (3, 22). Because acrosomes are adversely affected by thawing, it is speculated that acrosomal caps might become damaged during thawing of buffalo spermatozoa, as demonstrated in bull and rabbit sperm (6, 22). According to resultsin the present study, proportion of intact acrosome (Table 3) did not differcompared to thawed either for 37 or 30 seconds (p<0.05), and dramatically decreased during incubation for 2 hours for all groups.

However, Ansari et al (3) reported a higher post-thaw recovery of viable buffalo spermatozoa may be obtained in 0.25 ml straws by optimizing cooling procedures, rapid thawing and handling techniques compared to 0.5 ml straws (3). In the current study, the overall loss of normal acrosome during cryopreservation and thawing was less than 20%. This was lower than reported previously for buffalo bulls (6). This might be due to the use of a different extender, programmable freezing, or both, instead of the conventional ones.

The objective of this study was to use the Sperm Chromatin Structure Assay to determine the level and variability of damage of sperm DNA integrity in different thawing rates and incubation for 2 hours. Percentage of spermatozoa with high DNA fragmentation index (DFI) was significantly higher in the group inthawing was performed at 70℃at 6 seconds compared to other groups (Table 3). Incubation for 2 hours did not significantly increase % DFI (p<0.05). A similar result was found by Kadirvel et al. (21) who reported an overall mean DNA damage of 10.4% (range 4.8-17.6) for buffalo frozen-thawed sperm. They showed that in contrast with motility, the DNA integrity of spermatozoa remained unaffected during the freezing and thawing process (21). Abnormalities of chromatin structure and DNA integrity in mammalian sperm have been documented during low temperature storage (29). Percentages of post thaw spermatozoa with DFI in our study were relatively low, but in spite of these results there was evidence that fertilizing ability in bulldecreased with increased percentage of spermatozoa with DFI (25). A threshold of >30% DFI was statistically derived for significant lack of fertility potential in humans (12). Larson-Cook et al. (2) observed significant decrease in fertility if the percentage of spermatozoa with DFI exceeded 27% (2). In another study in boar chromatin was significantly more compact when thawing was performed at 50℃, but its stability did not show any difference relative to thawing at 42℃ in boars (5).

It is known that sperm chromatin damage/abnormal structure may be also caused by environmental factors such as elevated body temperature (12), toxic agents, components of the extender in which semen is stored, storage conditions or, in some species, technological procedures to which the semen is subjected (25, 29). Although the mechanisms responsible for increased nuclear DNA damage in spermatozoa are poorly understood, a potential explanation could be oxidative stress, since excessive reactive oxygen species can induce sperm DNA damage (29).

## Conclusion

In the present study, proportion of progressive motility and kinematic parameters such as of linear motility of spermatozoa was significantly increased by thawing at 37℃ in 30 seconds to 70℃ in 6 seconds. However, this relative advantage had disappeared after incubation in a water bath at 37℃ for two hours. Sperm thawing over at 50 degrees could be safely used to improve motility recovery after sperm cryopreservation in buffalo bulls. Further studies will be necessary to evaluate the possible clinical applications in buffalo.
